# The Interaction between Childhood Bullying and the *FKBP5* Gene on Psychotic-Like Experiences and Stress Reactivity in Real Life

**DOI:** 10.1371/journal.pone.0158809

**Published:** 2016-07-07

**Authors:** Paula Cristóbal-Narváez, Tamara Sheinbaum, Araceli Rosa, Sergi Ballespí, Marta de Castro-Catala, Elionora Peña, Thomas R. Kwapil, Neus Barrantes-Vidal

**Affiliations:** 1 Departament de Psicologia Clínica i de la Salut, Universitat Autònoma de Barcelona (UAB), Barcelona, Spain; 2 Secció de Zoologia i Antropologia, Departament de Biologia Evolutiva, Ecologia i Ciències Ambientals, Facultat de Biologia, Universitat de Barcelona (UB), Barcelona, Spain; 3 Centre for Biomedical Research Network on Mental Health (CIBERSAM), Instituto de Salud Carlos III, Madrid, Spain; 4 Institut de Biomedicina de la Universitat de Barcelona (IBUB), Barcelona, Spain; 5 Department of Psychology, University of North Carolina at Greensboro (UNCG), Greensboro, NC, United States of America; 6 Sant Pere Claver–Fundació Sanitària, Barcelona, Spain; Katholieke Universiteit Leuven, BELGIUM

## Abstract

**Aim:**

The present study employed Experience Sampling Methodology to examine whether the interaction between childhood bullying and *FKBP5* variability (i) is associated with the expression of psychotic-like experiences, paranoia, and negative affect, and (ii) moderates psychotic-like, paranoid, and affective reactivity to different forms of momentary stress (situational and social) in daily life.

**Methods:**

A total of 206 nonclinical young adults were interviewed for bullying with the Childhood Experience of Care and Abuse and were prompted randomly eight times daily for one week to complete assessments of their current experiences, affect, and stress appraisals. Participants were genotyped for three *FKBP5* single nucleotide polymorphisms (SNPs) (rs3800373, rs9296158, and rs1360780) that have been linked to hypothalamus-pituitary-adrenal axis reactivity. Multilevel analyses were conducted to examine the effect of the interaction between childhood bullying and the *FKBP5* haplotype derived from these three SNPs.

**Results:**

The interaction between bullying and the *FKBP5* haplotype was associated with positive, but not negative, psychotic-like experiences, paranoia, and negative affect. The bullying x *FKBP5* interaction also moderated the association of a social stress appraisal (specifically, being alone because people do not want to be with you) with psychotic-like experiences and negative affect in daily life. Simple slopes analyses indicated that, in all cases, the associations were significantly increased by exposure to bullying in participants with the risk haplotype, but not for those with the non-risk haplotype.

**Discussion:**

The present study provides the first evidence of the interplay between childhood bullying and *FKBP5* variability in the real-world expression of psychosis proneness and social stress reactivity. The findings underscore the importance of investigating how gene-environment interactions are involved in mechanistic pathways to the extended psychosis phenotype and lend further support to the increasing relevance given to socially defeating appraisals in the experience of reality distortion.

## Introduction

Mounting evidence indicates that childhood adversity is associated with an increased risk for psychosis phenotypes [1]. The association has been repeatedly observed with psychotic disorders, subclinical psychotic symptoms, and schizotypy traits [2–4]—consistent with converging research supporting the notion of etiological continuity between the clinical and nonclinical manifestations of psychosis [5, 6]. Among different types of interpersonal childhood adversity, experiences within the family milieu have been more extensively studied. However, there is increasing recognition that peer relations are key to children’s and adolescent’s developmental outcomes [7] and that exposure to bullying by peers can have a host of long-term detrimental effects [8]. Bullying involves intentional aggressive/hostile behavior in the context of actual or perceived imbalance of power [9, 10] and recent findings across different study designs (including prospective studies) and populations have demonstrated its association with psychotic phenomena [11–14].

It has been proposed that exposure to childhood interpersonal adversities may increase the risk for psychosis through a process of behavioral and biological sensitization involving hypothalamus-pituitary-adrenal (HPA) axis dysregulation and contributing to a final common pathway of dopamine sensitization in mesolimbic regions [15]. Dysregulation of the HPA axis involves dysregulation of the hypothalamic peptides of the corticotropin-releasing and arginine vasopressin hormones, resulting in enhanced release of plasma adrenocorticotropic hormone and glucocorticoid cortisol. Glucocorticoids promote the physiological stress response of fight-or-flight and are crucial for terminating the response through a negative feedback loop [16]. Such impaired negative feedback regulation via the glucocorticoid receptor (GR) has been proposed as a potential risk factor for stress-related psychopathology [16]. A critical regulator of GR activity is the FK506 binding protein 5 (FKBP5), a 51-kDa protein encoded by the *FKBP5* gene (located on chromosome 6p21.31 in humans) [17]. Notably, a functional haplotype that comprises up to 18 single nucleotide polymorphisms (SNPs) in the *FKBP5* gene has been related to increased expression of FKBP5 in response to GR activation and variation in GR sensitivity [16–18]. This haplotype is tagged by three SNPs (rs3800373, rs9296158, and rs1360780) that, for this reason, have been the most studied and characterized *FKBP5* polymorphisms [19]. Several studies pointed out that the risk alleles of these polymorphisms are the C, A, and T alleles, respectively [17]. Research has also suggested that the rs1360780 is the variant most likely conferring the haplotype functionality [17].

An increasing number of gene-environment interaction (GxE) studies have investigated the interaction between psychosocial stressors and *FKBP5* variability, with findings suggesting that adverse childhood experiences in interaction with the above mentioned haplotype are associated with risk for a range of psychopathological phenotypes (for review, see [17]). In this regard, recent studies showed that genetic variation in the *FKBP5* gene interacted with childhood trauma in the expression of psychotic phenomena in clinical and nonclinical samples [20–22]. Nevertheless, to the best of our knowledge, the role of the interaction between *FKBP5* variability and childhood bullying in particular has not been previously investigated.

There are also no GxE studies examining whether the interaction between childhood adverse experiences and *FKBP5* variability plays a role in heightening affective, paranoid, and psychotic-like responses to stress in daily life. Researchers have increasingly employed momentary assessment strategies to examine with ecological validity the experience and expression of psychological constructs in daily life as well as their environmental triggers (e.g., [23, 24]). The Experience Sampling Method (ESM) is a within-day self-assessment technique that prompts participants at random intervals to complete brief questionnaires about their current experiences, including stress, cognition, affect, and symptoms. By assessing participants in real time and in their real-life settings, ESM offers several advantages in comparison to traditional assessment techniques. These include the minimization of retrospective bias, enhanced ecological validity, and the ability to capture the context in which experiences occur [23, 25]. ESM measures exhibit good psychometric properties and have proven useful for examining the phenomenology and stress reactivity dynamics of the clinical and subclinical psychosis phenotypes [26–30]. In addition, it has been highlighted that utilizing prospective and repeated assessments of environmental exposures increases precision and reliability in the realm of GxE research [31]. Thus, the features of ESM data should enhance the power and quality of GxE studies and increase mechanistic insights that complement findings from large-scale epidemiological investigations [32–35].

In a previous ESM study we found that bullying was associated with psychotic-like experiences (PLEs) in daily life as well as with increased affective and paranoid reactivity to daily life stressors [36]. However, that study did not examine what factors may interact with bullying in shaping the expression of psychotic phenomena and stress reactivity in daily life. Therefore, the present study sought to examine in a non-clinically ascertained sample of young adults whether the interaction between bullying and *FKBP5* (i) is associated with PLEs, paranoia, and negative affect, and (ii) moderates psychotic-like, paranoid, and affective reactivity to different forms of momentary stress (i.e., situational and social) in daily life. We predicted that the interaction between bullying and the CAT risk haplotype of the *FKBP5* gene would be associated with higher levels of PLEs, paranoia, and negative affect, but not negative-like symptoms. We also expected that the previously reported association of stress with PLEs, paranoia, and affective experiences in daily life [26], and particularly the association with social stress, would be moderated by the bullying x *FKBP5* interaction, such that the association would be stronger for risk haplotype participants with childhood bullying exposure.

## Methods

### Ethics Statement

The present study was approved by the Universitat Autònoma de Barcelona Ethics Committee and conformed to the Helsinki Declaration. Participants had full capacity to consent to participation in research and gave written informed consent before taking part in the study.

### Participants

The sample forms part of PSYRIS-Barcelona, a longitudinal study examining psychosis risk and resilience. The sample was comprised of 206 nonclinically ascertained young adults from whom usable interview, ESM, and *FKBP5* genotype data were obtained. The participants were drawn from a screening sample of 589 undergraduate students (547 had complete usable data) at the Universitat Autònoma de Barcelona (Spain). Participants with high schizotypy scores were oversampled in order to ensure adequate representation of schizotypy in the current sample. A detailed description of the sample selection procedure has been provided elsewhere [26, 37]. The mean age of the sample was 21.3 years (*SD* = 2.4) and 78.6% were women. Ninety two percent were of European origin (subjects and both parents born in a European country).

### Materials and Procedure

#### Bullying

Questions from the Childhood Experience of Care and Abuse (CECA) [38] were used to assess bullying by peers. The CECA is a retrospective investigator-based interview that measures childhood experiences prior to the age of 17 years. Bullying is scored on a 4-point scale ranging from “marked” to “little/none”, based on specific rating rules and benchmarked thresholds. The interviews were conducted by psychologists and advanced graduate students in clinical psychology. Consensus meetings to discuss ratings were held regularly throughout the data collection period. The continuous severity ratings of bullying victimization were used for analyses.

#### ESM assessments

The ESM data collection was conducted with personal digital assistants (PDAs) that signaled participants randomly 8 times a day (between 10 a.m. and 10 p.m.) for one week to complete short questionnaires. When participants were signaled by the PDA, they had 5 minutes to initiate responding. After this time interval or the completion of the questionnaire, the PDA shut down until the next signal. The full list of ESM items can be found in Barrantes-Vidal et al. [26]. The social contact item was answered dichotmously (alone/with others), whereas the remaining items employed in the present study were answered on 7-point scales from “not at all” to “very much”.

ESM measures of symptoms, negative affect, social contact, and stress were used for analyses. Following Barrantes-Vidal et al. [26], we created indices of PLEs (8 items: unusual senses, unusual thoughts, feeling weird, losing control, difficulty controlling thoughts, familiar things seeming strange, hearing/seeing things others could not, and passivity; alpha index = .74) and paranoia (2 items: feeling suspicious and mistreated; alpha index = .70), and used the experience of diminished thoughts/emotions (“Right now I have no thoughts or emotions”) as a measure of negative-like symptoms. Negative affect was measured by an index composed of 4 items (feeling anxious, sad, angry, and guilty; alpha index = .80). With regard to stress, note that consistent with previous ESM research (e.g., [27, 30, 39, 40]), the present study did not focus on objective environmental stressors but rather on subjective appraisals of stress in daily life. The item “My current situation is stressful” was used to assess situational stress. Regarding social stress appraisals, we distinguished between social stress when participants were alone (assessed by the item “I am alone because people do not want to be with me”) and social stress when participants were with others (assessed by 2 items: “I feel close to this person (people)” and “Right now I would prefer to be alone”). Additionally, the social contact item was included in the analyses in order to distinguish the effects of social stress appraisals from the effects of simply being alone or with others at the time of the signal.

#### Genotyping

Genomic DNA was extracted using the Real Extraction DNA kit (Durviz S.L.U., Valencia, Spain). The three *FKBP5* SNPs rs3800373, rs9296158, and rs1360780 were genotyped using TaqMan 5’-exonuclease allelic discrimination assay (Applied Biosystems, custom assays: C_27489960_10, C_1256775_10, and C_8852038_10, respectively). Minor allele frequencies were 0.34 for rs3800373 (allele C), 0.33 for rs9296158 (allele A), and 0.33 for rs1360780 (allele T). No differences were observed between the allele frequencies in our sample and those reported in European (EUR from 1000 genomes) and Spanish (IBS from HapMap) reference populations (p > 0.05). Genotypic frequencies for each polymorphism are reported in Table 1. All SNPs were in accordance with Hardy-Weinberg Equilibrium (all *p* > 0.5).

**Table 1:** Description of the *FKBP5* Single Nucleotide Polymorphisms (SNPs) and Haplotype Groups (n = 206).

SNPs	Frequencies		Empirical background^a^	
Reference sequence (rs)	Genotypes	n (%)	Risk allele	Non-risk allele
rs3800373	C/C	24 (11.6%)	C	A
	A/C	92 (44.7%)		
	A/A	90 (43.7%)		
rs9296158	A/A	21 (10.2%)	A	G
	G/A	94 (45.6%)		
	G/G	91 (44.2%)		
rs1360780	T/T	24 (11.7%)	T	C
	C/T	88 (42.7%)		
	C/C	94 (45.6%)		
**Haplotype groups (n)**	Haplotypic combinations	n (%)	Risk haplotype	Non-risk haplotype
Risk carriers (n = 102)	CAT/CAT or CAT/XXX^c^	24 (11.6%)	CAT	AGC
	CAT/AGC	78 (37.9%)		
Non-risk carriers^b^ (n = 104)	AGC/AGC or AGC/XXX^c^	98 (47.6%)		
	XXX/XXX^c^	6 (2.9%)		

^a^ Risk and non-risk alleles according to previous studies.

^b^ Note that CAT/AGC combination has been included in the “risk carriers” group.

^c^ XXX = Other haplotype combinations (AAC, AAT, CGC, CGT, CAC, or AGT).

Linkage disequilibrium, which is the tendency of SNPs to be inherited together, was examined by pair-wise comparisons of r^2^ and D’using Haploview version 4.2 [41]. The three studied SNPs were observed to be in high linkage disequilibrium (D’ = 0.89). Haplotypes considering these three polymorphisms were estimated using a bayesian approach implemented with PHASE software [42]. The frequencies of the CAT (risk haplotype) and the AGC (non-risk haplotype) were 0.29 and 0.62, respectively. For analyses, participants were classified in two groups: i) risk carriers, which included carriers of at least one risk haplotype (i.e., CAT/CAT, CAT/XXX, or CAT/AGC), and ii) non-risk carriers, which included non-carriers of the risk haplotype (i.e., AGC/AGC, AGC/XXX, or XXX/XXX) (Table 1).

### Statistical Method

ESM data have a hierarchical structure in which ESM ratings (level 1 data) are nested within participants (level 2 data). Multilevel or hierarchical linear modeling provides a more appropriate method than conventional unilevel analyses for analyzing nested data and is standard for the analysis of ESM data [43, 44].

Two types of multilevel analyses were conducted in the present study. First, in order to examine the impact of the interaction of bullying and *FKBP5* on PLEs, paranoia, and negative affect in daily life, we assessed the independent effect of level 2 predictors (bullying, *FKBP5*, and the bullying x *FKBP5* interaction) on level 1 dependent measures (ESM ratings in daily life). Note that as described above we have already reported the association of bullying with ESM ratings in a study examining a wide variety of adversity exposures on daily-life experiences [36]; therefore, the main effects of bullying are not the object of the current study and will be solely described as a necessary step required to yield the GxE interaction. Second, to analyze whether the bullying x *FKBP5* interaction moderates the association of momentary stress with experiences in daily life, cross-level interactions (or slopes-as-outcomes) were computed. Cross-level interactions were used to examine whether level 1 relationships (e.g., the association between feeling unwanted and PLEs in daily life) vary as a function of level 2 variables (e.g., the bullying x *FKBP5* interaction). In both the analyses of direct effects and cross-level interactions, the effect of bullying and the *FKBP5* haplotype were examined separately (i.e., two separate models were used, one in which bullying was the predictor and another in which the *FKBP5* haplotype was the predictor).

In order to examine the effect of the bullying x *FKBP5* interaction, bullying, the *FKBP5* haplotype, and the interaction term were entered simultaneously in the same model. When a significant bullying x *FKBP5* interaction was found, the effect of the interaction was examined within each haplotype group using simple slopes. The multilevel analyses were computed with MPlus 6 [45]. Graphics and simple slopes were computed with HLM 7.01 program [46]. Level 1 predictors were group mean centered and level 2 predictors were grand mean centered. The data departed from normality in some cases, so parameter estimates were calculated using robust standard errors. Furthermore, level 1 criteria exhibiting significant skew were treated as categorical.

## Results

Participants completed an average of 40.8 usable ESM questionnaires (*SD* = 9.1). The *FKBP5* risk haplotype and bullying were not correlated (*r* = 0.08) and neither was associated with the number of usable records (*r* = 0.03 and -0.04, respectively). Additionally, there were no sex differences in either variable (bullying: *t* = 0.849, *p* = 0.397; *FKBP5* haplotype: χ² = 1.658, *p* = 0.198).

As shown in Table 2, bullying was associated with PLEs and negative affect but not with paranoia or negative-like symptoms. The *FKBP5* haplotype was not associated with daily life symptoms or negative affect. The interaction of bullying and the *FKBP5* haplotype was significantly associated with PLEs, paranoia, and negative affect. Simple slopes analyses indicated that, as expected, bullying increased PLEs and paranoia for participants with the risk haplotype (0.059, *SE* = 0.021, *t* = 2.78, *p* < 0.01; 0.085, *SE* = 0.035, *t* = 2.41, *p* < 0.05, respectively), but not for those with the non-risk haplotype (0.002, *SE* = 0.019, *t* = 0.08, ns; -0.018, *SE* = 0.031, *t* = -0.58, ns, respectively). Similarly, bullying was associated with increased negative affect for participants with the risk haplotype (0.188, *SE* = 0.051, *t* = 3.72, *p* < 0.001), but not for those with the non-risk haplotype (0.037, *SE* = 0.058, *t* = 0.64, ns).

**Table 2:** Main Effects of Bullying, the *FKBP5* Haplotype, and their Interaction on Psychosis Spectrum Experiences and Negative Affect (n = 206).

Level 1 Criterion	Level 2 Predictors		
	Bullying	*FKBP5*	Bullying x *FKBP5*^b^
	γ_01_ (*df* = 204)	γ_01_ (*df* = 204)	γ_03_ (*df* = 202)
**Psychosis Spectrum**			
Psychotic-like index	0.034 (SE = 0.015)*	-0.008 (SE = 0.022)	0.027 (SE = 0.013)*
Paranoia index	0.038 (SE = 0.026)	-0.053 (SE = 0.047)	0.048 (SE = 0.022)*
No thoughts/emotions^a^	0.289 (SE = 0.168)	-0.299 (SE = 0.329)	0.199 (SE = 0.162)
**Affect**			
Negative affect index	0.113 (SE = 0.040)**	-0.128 (SE = 0.067)	0.071 (SE = 0.036)*

* *p* < .05

** *p* < .01.

^a^ Item was run as categorical.

^b^ Bullying and *FKBP5* were examined independently. The interaction was examined with bullying and *FKBP5* in the model.

**Fig 1:**
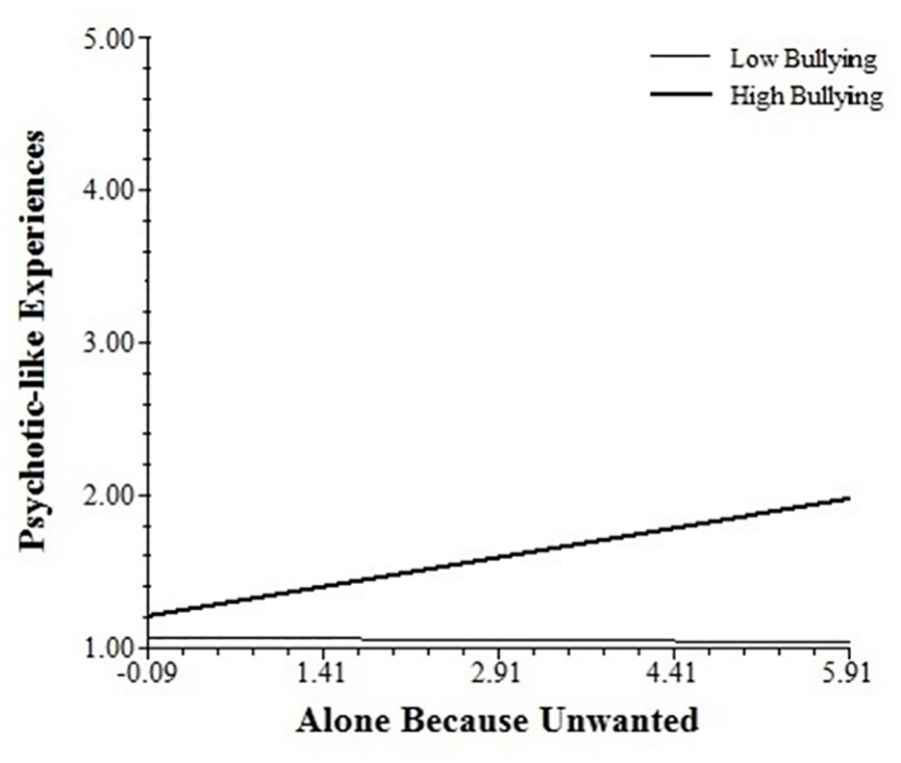
Association between feeling unwanted and PLEs across levels of bullying in *FKBP5* risk-haplotype participants.

Cross-level interaction analyses examined whether bullying, the *FKBP5* haplotype, and their interaction moderated the association of social contact and stress appraisals with PLEs, paranoia, and negative affect in daily life (Table 3). Bullying moderated the association of situational stress and preference to be alone with paranoia. It also moderated the association of social contact with PLEs and that of decreased social closeness with negative affect. The *FKBP5* haplotype did not moderate the associations of situational stress or social stress with experiences in daily life. The bullying x *FKBP5* interaction moderated the association of feeling unwanted when alone and PLEs in daily life. Simple slopes analyses indicated that the association between feeling unwanted and PLEs was significantly increased by exposure to bullying in participants with the risk haplotype (0.056, *SE* = 0.027, *t* = 2.07, *p* < 0.05; see Fig 1), but not for those with the non-risk haplotype (-0.034, *SE* = 0.020, *t* = -1.74, ns).

**Table 3:** Cross-Level Interactions with Bullying, the *FKBP5* Haplotype, and the Bullying x *FKBP5* Interaction (n = 206).

Level 1 Criterion	Level 1 Predictor^a^		Level 2 Predictors		
			Bullying	*FKBP5*	Bullying x *FKBP5*^b^
	γ_10_ (*df* = 204)		γ_11_ (*df* = 204)	γ_11_ (*df* = 204)	γ_13_ *(df* = 202)
Psychotic-like index	Situation stressful	0.035 (SE = 0.004)***	0.006 (SE = 0.006)	0.002 (SE = 0.009)	0.004 (SE = 0.005)
Paranoia index	Situation stressful	0.078 (SE = 0.009)***	0.029 (SE = 0.012)*	0.015 (SE = 0.020)	0.017 (SE = 0.011)
Negative affect index	Situation stressful	0.215 (SE = 0.012)***	0.015 (SE = 0.012)	-0.001 (SE = 0.023)	0.005 (SE = 0.012)
Psychotic-like index	Alone	0.000 (SE = 0.006)	-0.015 (SE = 0.006)*	-0.008 (SE = 0.012)	-0.006 (SE = 0.005)
Paranoia index	Alone	-0.008 (SE = 0.014)	0.001 (SE = 0.014)	-0.030 (SE = 0.028)	-0.001 (SE = 0.014)
Negative affect index	Alone	-0.046 (SE = 0.018)*	0.012 (SE = 0.018)	0.042 (SE = 0.035)	0.015 (SE = 0.018)
Psychotic-like index	Alone b/c not wanted	0.083 (SE = 0.018)***	0.019 (SE = 0.023)	0.001 (SE = 0.040)	0.037 (SE = 0.016)*
Paranoia index	Alone b/c not wanted	0.150 (SE = 0.048)**	0.039 (SE = 0.053)	-0.029 (SE = 0.108)	0.054 (SE = 0.044)
Negative affect index	Alone b/c not wanted	0.170 (SE = 0.046)***	0.075 (SE = 0.044)	0.150 (SE = 0.104)	0.104 (SE = 0.044)*
Psychotic-like index	Close to other	-0.009 (SE = 0.003)**	-0.004 (SE = 0.003)	0.005 (SE = 0.005)	-0.002 (SE = 0.003)
Paranoia index	Close to other	-0.027 (SE = 0.008)***	-0.017 (SE = 0.009)	0.016 (SE = 0.015)	-0.005 (SE = 0.008)
Negative affect index	Close to other	-0.048 (SE = 0.009)***	-0.022 (SE = 0.009)*	0.008 (SE = 0.017)	-0.001 (SE = 0.009)
Psychotic-like index	Prefer to be alone	0.020 (SE = 0.004)***	0.004 (SE = 0.005)	0.009 (SE = 0.009)	0.004 (SE = 0.004)
Paranoia index	Prefer to be alone	0.070 (SE = 0.010)***	0.028 (SE = 0.014)*	0.007 (SE = 0.022)	0.019 (SE = 0.012)
Negative affect index	Prefer to be alone	0.126 (SE = 0.013)***	0.024 (SE = 0.014)	-0.012 (SE = 0.026)	0.013 (SE = 0.013)

* *p* < .05

** *p* < .01

*** *p* < .001.

^a^ Note that the statistical significance of the associations of the level 1 predictor and criterion did not vary across each level 2 predictor. The table reports the coefficient of the association of the level 1 predictor and criterion for the analyses of bullying.

^b^ Bullying and *FKBP5* were examined independently. The interaction was examined with bullying and *FKBP5* in the model.

**Fig 2:**
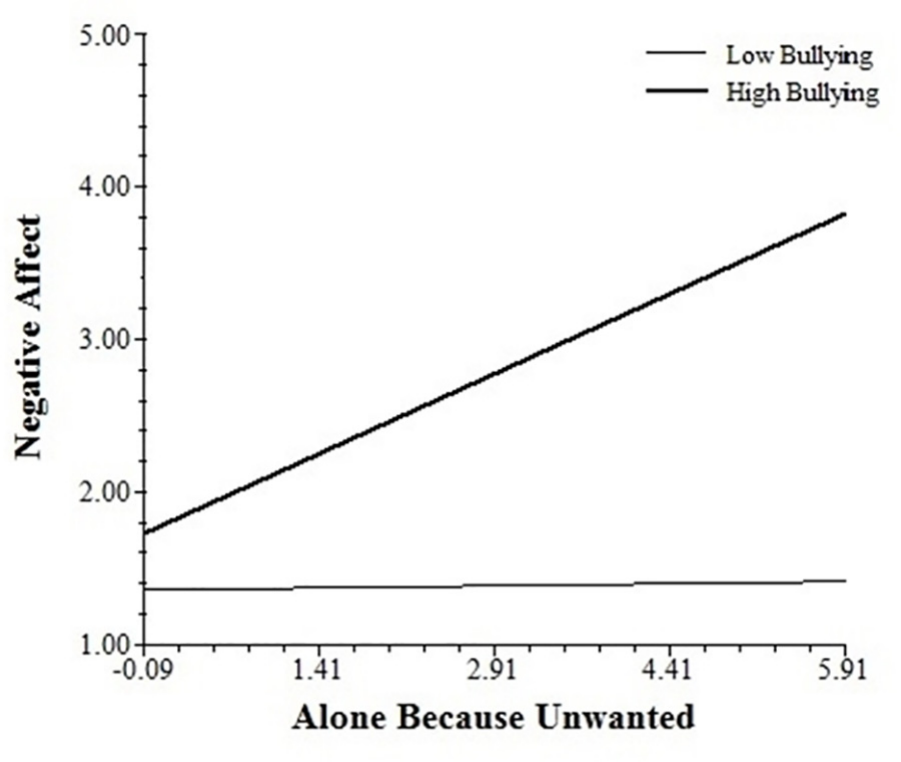
Association between feeling unwanted and negative affect across levels of bullying in *FKBP5* risk-haplotype participants.

The bullying x *FKBP5* interaction also moderated the association of feeling unwanted with negative affect. Simple slopes analyses showed that the association between this appraisal and negative affect was significantly increased by exposure to bullying in risk haplotype participants (0.144, *SE* = 0.051, *t* = 2.80, *p* < 0.01; see Fig 2), but not in non-risk haplotype participants (-0.092, *SE* = 0.075, *t* = -1.23, ns).

### Additional Analyses

Following the recommendation of a reviewer, we performed exploratory analyses partialing out the CECA parental antipathy ratings to examine whether the effects of the bullying x *FKBP5* interaction on daily life outcomes were found over-and-above the effects of another form of childhood maltreatment. Specifically, we partialed the parental antipathy score out of the main effects of bullying and *FKBP5*, and the antipathy rating and the antipathy x bullying and antipathy x *FKBP5* interaction out of the analysis of the bullying x *FKBP5* interaction. This followed the reviewer's suggestion that we select a single CECA exposure to use as a confounding measure. For this sample CECA parental antipathy and role reversal exposures were available (peak rating taking into account behavior of mother and father figure). We selected parental antipathy because (i) it shares similarities with peer bullying (e.g., involving rejection, coldness, hostility) and (ii) it is carried out by different figures than peer bullying. Note that parental antipathy had a modest (although not significant) correlation with bullying in our sample (r = .13)–making it an ideal covariate. This method follows recommendations by Keller [47], who suggested that in order to properly control for potential confounders, all the covariate-by-environment and the covariate-by-gene interaction terms should be entered in the same model that tests the gene-by-environment interaction term. However, it should be interpreted cautiously in the current study because of the post hoc nature of the analyses and the lack of a priori selection of parental antipathy. We reran all of the analyses in Tables 1 and 2 using this strategy. The results were largely unchanged (see S1 and S2 Tables). All the significant bullying x *FKBP5* interactions remained, and the association between situational stress and paranoia became significantly moderated by the bullying x *FKBP5* interaction. Simple slopes indicated that the association was significantly increased by exposure to bullying in participants with the risk haplotype (0.044, *SE* = 0.018, *t = 2*.46, *p* < 0.05), but not for those with the non-risk haplotype (0.007, *SE* = 0.014, *t =* 0.48, ns). These additional analyses add support to the interpretation that the bullying x *FKBP5* effects are robustly significant and attributable to this particular type of adversity.

## Discussion

To the best of our knowledge, the current study is the first to examine the interplay between bullying and *FKBP5* variability in the expression of psychotic phenomena and stress reactivity in the realm of daily life. The results indicated that the interaction between bullying and the risk *FKBP5* haplotype was associated with PLEs, paranoia, and negative affect, and that it moderated psychotic-like and affective reactivity to a social stress appraisal (i.e., feeling unwanted by others) in a nonclinical sample. This work expands on previous GxE research supporting that the interaction between *FKBP5* variability and childhood adversity exposure increases the risk for psychosis phenotypes. The findings contribute to our understanding of how the complex interplay between genetic and environmental factors is involved in the real-world expression of psychosis proneness.

The results regarding the interaction between bullying and *FKBP5* variability on psychotic-like, paranoid, and affective experiences were in line with our hypotheses and provide evidence of a GxE interaction on subclinical psychotic phenomena in real life. Furthermore, the finding that the interaction was not associated with negative-like symptoms is consistent with the contention that positive and negative psychotic features may involve different etiological pathways [48], with environmental adversity exposures and biological mechanisms involved in regulating the stress response thought to be particularly relevant for the positive symptom dimension (e.g., [15]).

Prior research has consistently shown that exposure to interpersonal childhood adversities increases the risk for several psychopathological phenotypes in carriers of the functional haplotype associated with higher FKBP5 induction and prolonged cortisol responses [17]. Although next-generation sequencing projects have enabled to catalogue the broad range of variants in the *FKBP5* gene (e.g., [49]), the majority of these studies have investigated *FKBP5* variability using a tagging approach and focusing on some of the most common tag SNPs (rs3800373, rs9296158, or rs1360780) of this haplotype [18]. However, the investigated SNPs have not been the same in all studies and, to our knowledge, there are no studies examining specifically the role of the haplotype comprised by these three tag SNPs on psychosis proneness. Nevertheless, the finding that the bullying and *FKBP5* interaction was associated with positive psychotic phenomena is consistent with a recent study showing that childhood abuse was associated with increased PLEs in carriers of the risk alleles of rs1360780 in a nonclinical sample [22]. Our results are also in agreement with the first study examining the role of *FKBP5* in psychosis [20], which found that carriers of the rs1360780 and rs9296158 risk alleles (as well as rs1043805, which was not investigated here) were more vulnerable to the effect of childhood trauma on PLEs in a general population sample. In the same study, they also found that rs9296158 moderated the effect of trauma on psychotic symptoms in patients with a psychotic disorder. Of note, neither our study nor previous ones in nonclinical and clinical samples [20–22, 50] found that *FKBP5* variability by itself was associated with positive psychotic phenomena or presence of a psychotic disorder. Therefore, taken together, findings are in line with the notion that the contribution of *FKBP5* variability to psychosis risk may be dependent upon the presence of specific environmental exposures [51].

The finding that the association of the risk alleles of the haplotype with psychotic phenomena is commonly triggered by exposure to childhood adversity is interesting in light of recent molecular studies suggesting that childhood trauma exposure could induce allele-specific epigenetic modifications that may increase the risk for stress-related phenotypes [52]. Specifically, Klengel et al. [52] found that childhood abuse exposure was associated with preferential demethylation of DNA (near a glucocorticoid response element in *FKBP5*) in risk allele carriers, which enhances differences in glucocorticoid receptor sensitivity and entails a dysregulation of the stress system that may eventually increase vulnerability for certain psychopathological phenotypes. Importantly, this reduced methylation seemed to be dependent specifically on childhood abuse exposure, but not adult trauma exposure or current levels of cortisol, indicating that there may be a critical developmental stage for such epigenetic effects [52].

Regarding stress reactivity, we found that the GxE interaction moderated the association of appraisals of being unwanted when alone with PLEs and negative affect. In particular, our results indicated that these associations were significantly increased by exposure to bullying in risk haplotype participants, but not in non-risk haplotype participants. By contrast, the interaction did not moderate affective and symptomatic reactivity to situational stress and other forms of social stress (i.e., appraisals of diminished closeness and increased preference for being alone). In light of these results, it is attractive to speculate that social defeat is a mechanism involved in increasing reactivity in individuals with the risk haplotype.

More specifically, it has been suggested that childhood adversity may increase psychosis vulnerability by inducing a state of social defeat, characterized by feelings of outsider status and decreased self-value [53, 54]. Of note, recent research has indicated that social defeat plays a mediating role in the association between childhood trauma and psychotic phenotypes at the population level [55], and that a history of social defeat increases the likelihood of psychotic responses during social interactions in an experimental social environment generated by Virtual Reality in clinically at-risk individuals [56]. Bullying has been conceptualized as a socially defeating experience and its parallels with animal models of social defeat have been highlighted [57]. Likewise, the appraisal of being alone because others do not want to be with you could be considered a proximal micro-level experience of social defeat. Previous work indicated that mice lacking the *FKBP5* gene showed decreased neuroendocrine/physiological responses to chronic social defeat stress (as compared with wild-type animals), pointing to an increased glucocorticoid negative feedback of the HPA axis that may be modulated by heightened GR sensitivity [58]. Such findings support human studies suggesting that *FKBP5* risk alleles may increase sensitivity to psychosocial adversities through an enhanced FKBP5 expression and thereby diminished GR sensitivity [16, 17]. In this context, our results may therefore suggest that the *FKBP5* risk haplotype amplifies the likelihood that distal experiences of social defeat will increase psychotic-like reactivity to proximal socially defeating appraisals.

Strengths of the present study include the use of an interview measure to assess bullying, which allowed to obtain in-depth information and minimize biases related to subjective responding [59]. The estimation of the risk haplotype is also a strength given that it reports the full variability of a DNA fragment and increases the power to find genetic associations [60]. In addition, we employed ecologically valid measures of experiences obtained prospectively and repeatedly during a one-week period, increasing the power and reliability of GxE research [31, 32]. Finally, although we computed multiple analyses, they were limited to a priori goals and hypotheses of the study to avoid exploratory analyses that would increase the risk of Type I error. Also, following the suggestion of a reviewer, we confirmed that the findings reported remain largely unchanged after partialing out the effect of another type of adversity (parental antipathy), which strengthens the role of bullying as a relevant exposure. Limitations of the study include the cross-sectional nature of the data, which limits interpretations about the causal effects of GxE interactions. Similarly, given that predictor and criterion ESM variables were measured concurrently, causal inferences regarding the effects of stress appraisals cannot be definitively made. Furthermore, the generalizability of the present results is limited by the use of a predominantly female university student sample. Future studies should investigate at-risk and clinical samples to identify whether the interaction between bullying and *FKBP5* variability is relevant across the psychosis continuum. Likewise, further research may consider assessing whether the direct and cross-level effects reported in the current study are found over-and-above the effects of other childhood adversity exposures.

To conclude, the present study provides a novel contribution by showing that bullying and the *FKBP5* risk haplotype interact in shaping the expression of reality distortion and social stress reactivity in real life. The current study concurs with and expands previous work by providing evidence supporting the 3-hit [51] and sensitization [15] hypotheses, that is, the relevance of the interaction of 1) genetic risk, 2) distal environmental factors and 3) proximal environmental re-exposures on the expression of psychosis proneness. Our findings highlight that examining the interplay between genetic and environmental factors should increase our understanding of the mechanistic pathways leading to the extended psychosis phenotype and further support the increasing relevance given to socially defeating appraisals in the experience of reality distortion.

## Supporting Information

S1 Table Main Effects of Bullying, the *FKBP5* Haplotype, and their Interaction on Psychosis Spectrum Experiences and Negative Affect Partialing out the Effects of Parental Antipathy (n = 206). (DOCX)

S2 Table Cross-Level Interactions with Bullying, the *FKBP5* Haplotype, and the Bullying x *FKBP5* Interaction Partialing out the Effects of Parental Antipathy (n = 206). (DOCX)
