# Supplementary material for: The Interaction between Childhood Bullying and the FKBP5 Gene on Psychotic-Like Experiences and Stress Reactivity in Real Life
Source: PLoS One. 2016 Jul 7;11(7):e0158809. doi: 10.1371/journal.pone.0158809 (PMC4936666; doi:10.1371/journal.pone.0158809)
Supplement: S1 Table — (DOCX) [file pone.0158809.s001.docx]

**S1 Table. Main Effects of Bullying, the *FKBP5* Haplotype, and their Interaction on Psychosis Spectrum Experiences and Negative Affect Partialing out the Effects of Parental Antipathy (n=206)**

| **Level 1 Criterion** |  | |  | **Level 2 Predictors** | | |  |  |
| --- | --- | --- | --- | --- | --- | --- | --- | --- |
|  | Bullying^b^ | Parental  Antipathy^b^ | *FKBP5*^b^ | | Parental  Antipathy^b^ | Antipathy x Bullying^c^ | Antipathy x *FKBP5*^c^ | Bullying x *FKBP5*^c^ |
|  | γ_01_ (*df*=203) | γ_02_ (*df*=203) | γ_01_ (*df*=203) | | γ_02_ (*df*=203) | γ_04_ (*df*=199) | γ_05_ (*df*=199) | γ_06_ (*df*=199) |
|  | Coeff. (SE) | Coeff. (SE) | Coeff. (SE) | | Coeff. (SE) | Coeff. (SE) | Coeff. (SE) | Coeff. (SE) |
| **Psychosis Spectrum** |  |  |  | |  |  |  |  |
| Psychotic-like index | 0.032 (0.012)* | 0.021 (0.014) | -0.009 (0.022) | | 0.025 (0.013) | -0.021 (0.012) | -0.007 (0.011) | 0.030 (0.014)* |
| Paranoia index | 0.030 (0.028) | 0.063 (0.031)* | -0.056 (0.047) | | 0.068 (0.030)* | -0.042 (0.024) | -0.024 (0.024) | 0.053 (0.022)* |
| No thoughts/emotions^a^ | 0.281 (0.173) | 0.067 (0.170) | -0.309 (0.332) | | 0.114 (0.170) | -0.083 (0.181) | 0.095 (0.188) | 0.201 (0.175) |
| **Affect** |  |  |  | |  |  |  |  |
| Negative affect index | 0.107 (0.041)* | 0.049 (0.040) | -0.131 (0.067) | | 0.064 (0.039) | -0.050 (0.033) | 0.001 (0.033) | 0.076 (0.036)* |

**p* <.050, ***p* <.010, ****p* < .001. ^a^ Item was run as categorical. ^b^ The parental antipathy rating was partialed out of the main effects of bullying and *FKBP5,* which were examined independently. ^c^ In order to examine the effect of the bullying x *FKBP5* interaction, all simple effects (bullying, *FKBP5* haplotype, parental antipathy) and interaction effects between the covariate and the genetic and environmental variables (antipathy x bullying and antipathy x *FKBP5* haplotype) were entered in the same model.
