# Supplementary material for: The Interaction between Childhood Bullying and the FKBP5 Gene on Psychotic-Like Experiences and Stress Reactivity in Real Life
Source: PLoS One. 2016 Jul 7;11(7):e0158809. doi: 10.1371/journal.pone.0158809 (PMC4936666; doi:10.1371/journal.pone.0158809)
Supplement: S2 Table — (DOCX) [file pone.0158809.s002.docx]

**S2 Table. Cross-Level Interactions with Bullying, the *FKBP5* Haplotype, and the Bullying x *FKBP5* Interaction Partialing out the Effects of Parental Antipathy (n=206)**

| **Level 1 Criterion** | **Level 1 Predictor**^a^ | |  | | **Level 2 Predictors** | |  | | |
| --- | --- | --- | --- | --- | --- | --- | --- | --- | --- |
| Indices |  | | Bullying^b^ | Parental  Antipathy^b^ | *FKBP5*^b^ | Parental  Antipathy^b^ | Antipathy x Bullying^c^ | Antipathy x *FKBP5*^c^ | Bullying x *FKBP5*^c^ |
|  | γ_10_ (*df*=203) | | γ_11_ (*df*=203) | γ_12_ (*df*=203) | γ_11_ (*df*=203) | γ_12_ (*df*=203) | γ_14_ (*df*=199) | γ_15_ (*df*=199) | γ_16_ (*df*=199) |
|  |  | | Coeff. (SE) | Coeff. (SE) | Coeff. (SE) | Coeff. (SE) | Coeff. (SE) | Coeff. (SE) | Coeff. (SE) |
| Psychotic-like | Situation stressful | 0.035 (0.004)*** | 0.005 (0.006) | 0.007(0.005) | 0.001 (0.008) | 0.008 (0.005) | -0.005 (0.006) | 0.002 (0.004) | 0.005 (0.004) |
| Paranoia | Situation stressful | 0.078 (0.009)*** | 0.028 (0.013)* | 0.008 (0.013) | 0.014 (0.020) | 0.012 (0.012) | -0.027 (0.011)* | 0.007 (0.011) | 0.021 (0.010)* |
| Negative affect | Situation stressful | 0.215 (0.012)*** | 0.014 (0.012) | 0.006 (0.012) | -0.002 (0.023) | 0.008 (0.012) | -0.010 (0.011) | 0.013 (0.011) | 0.005 (0.012) |
| Psychotic-like | Alone | 0.000 (0.006) | -0.015 (0.006)* | 0.005 (0.008) | -0.008 (0.012) | 0.004 (0.008) | -0.008 (0.005) | 0.010 (0.006) | -0.007 (0.005) |
| Paranoia index | Alone | -0.008 (0.014) | -0.002 (0.015) | 0.029 (0.017) | -0.030 (0.027) | 0.029 (0.016) | -0.024 (0.012)* | -0.003 (0.015) | 0.001 (0.014) |
| Negative affect | Alone | -0.046 (0.018)* | 0.009 (.019) | 0.024 (0.019) | 0.041 (0.035) | 0.025 (0.018) | 0.007 (0.016) | 0.021 (0.018) | 0.010 (0.019) |
| Psychotic-like | Alone b/c not wanted | 0.085 (0.019)*** | 0.020 (0.023) | -0.011 (0.021) | 0.002 (0.039) | -0.010 (0.021) | -0.004 (0.017) | 0.006 (0.013) | 0.039 (0.017)* |
| Paranoia index | Alone b/c not wanted | 0.145 (0.047)** | 0.038 (0.054) | 0.019 (0.062) | -0.032 (0.110) | 0.024 (0.063) | -0.008 (0.045) | -0.042 (0.053) | 0.055 (0.045) |
| Negative affect | Alone b/c not wanted | 0.168 (0.051)** | 0.074 (0.043) | 0.009 (0.050) | 0.149 (0.102) | 0.010 (0.052) | 0.055 (0.041) | -0.053 (0.044) | 0.100 (0.042)* |
| Psychotic-like | Close to other | -0.009 (0.003)** | -0.003 (0.003) | -0.004 (0.003) | 0.006 (0.005) | -0.004 (0.003) | 0.002 (0.002) | 0.000 (0.003) | -0.002 (0.003) |
| Paranoia index | Close to other | -0.027 (0.007)*** | -0.016 (0.009) | -0.001 (0.010) | 0.017 (0.015) | -0.004 (0.010) | 0.000 (0.007) | -0.005 (0.008) | -0.004 (0.008) |
| Negative affect | Close to other | -0.048 (0.009)*** | -0.022 (0.010)* | -0.001(0.008) | 0.009 (0.017) | -0.004 (0.008) | -0.003 (0.007) | 0.004 (0.007) | -0.001 (0.010) |
| Psychotic-like | Prefer to be alone | 0.020 (0.004)*** | 0.004 (0.005) | 0.006 (0.005) | 0.008 (0.009) | 0.006 (0.005) | -0.003 (0.004) | 0.003 (0.004) | 0.004 (0.004) |
| Paranoia index | Prefer to be alone | 0.069 (0.010)*** | 0.026 (0.014) | 0.022 (0.013) | 0.003 (0.022) | 0.024 (0.013) | -0.003 (0.012) | 0.001 (0.010) | 0.019 (0.012) |
| Negative affect | Prefer to be alone | 0.126 (0.013)*** | 0.024 (0.014) | 0.001 (0.013) | -0.013 (0.026) | 0.003 (0.013) | -0.008 (0.011) | 0.010 (0.011) | 0.013 (0.013) |

**p* <.050, ***p* <.010, ****p* < .001. ^a^ Note that the statistical significance of the associations of the level 1 predictor and criterion did not vary across each level 2 predictor. The table reports the coefficient of the association of the level 1 predictor and criterion for the analyses of bullying. ^b^ The parental antipathy rating was partialed out of the main effects of bullying and *FKBP5,* which were examined independently. ^c^ In order to examine the effect of the bullying x *FKBP5* interaction, all simple effects (bullying, *FKBP5* haplotype, antipathy) and interaction effects between the covariate and the genetic and environmental variables (antipathy x bullying and antipathy x *FKBP5* haplotype) were entered in the same model.
